# Under-served groups’ perspectives on mitigating digital exclusion within healthcare in the North East of England

**DOI:** 10.1038/s44482-026-00022-w

**Published:** 2026-07-03

**Authors:** Sarah Wilson, Clare Tolley, Riona Mc Ardle, Nehal Hassan, Robert Slight, Sarah Slight

**Affiliations:** 1 Newcastle University, Newcastle Upon Tyne, UK; 2 Newcastle NIHR Biomedical Research Centre, Newcastle Upon Tyne, UK; 3 The Newcastle upon Tyne Hospitals NHS Foundation Trust, Newcastle Upon Tyne, UK

**Keywords:** Business and industry, Health care

## Abstract

Digital Health Technologies (DHTs) can increase access to healthcare and improve patient outcomes, but underserved groups often experience barriers accessing these benefits. This qualitative study explored the perspectives of individuals from underserved groups on using DHTs within healthcare and what practical strategies could be implemented to mitigate digital exclusion. Participants described difficulties accessing healthcare when it was only available digitally, specific challenges relating to DHTs, issues accessing technical support, and concerns about DHTs’ ability to support all areas of healthcare. Participants recommended that healthcare providers offer support such as free devices, maintain non-digital access options, and that DHTs are co-designed to be user-friendly. They also called for both increased funding for and better promotion of community-based technical support services, and appropriate user guides. Future research should explore stakeholders’ perspectives on the feasibility of implementing these recommendations to develop a holistic understanding of how to effectively support digital health equity.

## Introduction

Digital exclusion is defined as the marginalisation of an individual or group deprived of access and use of digital technologies^1^. Digital exclusion is caused by barriers accessing compatible devices and/or connectivity, an individual’s inability to use digital technologies, and/or their lack of motivation to use digital technologies^1–4^. As more public services are now delivered online, the numbers experiencing digital exclusion continues to grow^3^; estimates suggest that 2.9 billion people were digitally excluded worldwide in 2021^5^. Within the UK (United Kingdom), an estimated 10 million adults were digital excluded in 2019^6^, and it has been predicted that 5.8 million people will continue to remain digitally excluded in the UK by the end of 2032 if nothing is done to support them^7^.

Healthcare systems have promoted the use of digital health technologies (DHTs) within their services over recent years^8–11^. The National Health Service (NHS) in England has placed DHTs at the centre of their 10-year health plan, stating *“the NHS App will become the main way people organise and manage their care”*^12^. Examples of DHTs include telephone calls, apps, sensors, wearable devices, and online platforms^8,13^. However, health inequities will be exacerbated if the design, development and/or implementation of DHTs does not consider the needs of individuals experiencing digital exclusion^14^. Those most likely to experience health inequities are under-served groups, a term used in the UK to describe demographic groups typically marginalised within society due to cultural/social norms, stigmas, discrimination and power imbalances between social groups^15,16^. The needs of underserved groups have often been neglected in the development of DHTs, with a focus being on digitally literate and “able-bodied” individuals, consequently widening the “digital divide”^17,18^.

Various organisations and regulatory bodies have recognised the need to mitigate digital exclusion to reduce health inequities. This has led to changes on a global, national (UK) and regional scale, and influenced regulatory approval processes. Digital inclusion strategies have been developed and implemented sporadically across different communities. These included the provision of resources (e.g., devices or connectivity) to those who needed them, ensuring DHTs were appropriate (in terms of usability, affordability, culturally, and accessibility) for different underserved groups^9^, and providing technical support (from family, friends or professionals) to aid their use of DHTs^19–21^. There has been interest in tackling digital exclusion over recent years with published research exploring lived experiences of digital exclusion^22^, and identifying potential recommendations to support digital health equity^23,24^. However, there has been little consideration given to individuals who identify with two or more underserved groups at risk of digital exclusion (i.e., intersectionality). We believe our qualitative study is the first to explore underserved groups’ perspectives and experiences of using DHTs to gain a better understanding of how to mitigate digital exclusion within healthcare and support health equity.

## Results

### Participant characteristics

Twenty-nine individuals participated in either a focus group (*n* = 18) or semi-structured interview (*n* = 11). One participant withdrew on the day of the interview without reason. Four focus groups were conducted (with 3–6 participants) and lasted between 90–120 min, with a 5–10 min comfortable break offered if required. Eleven interviews were conducted and lasted between 40–60 min. Twenty participants were female and nine were male. All CLEARS groups were represented. Participants self-reported as representing one or more CLEARS subcategories. The cultural group with three subcategories (i.e., diverse ethnicity, English as a second language, and has a religious belief) was the most prevalent (*n* = 38) and the least represented group was limiting conditions with three subcategories (i.e., visual impairment, hearing impairment or both visual and hearing impairment) (*n* = 10) (Tables 1 and 2). Participants resided in a range of boroughs across the North East including North Tyneside, Newcastle, and South Tyneside. Most participants (*n* = 22) had not previously taken part in research, and all participants had access to at least one device (e.g., smartphone, mobile phone, smart tablets etc.). Many participants reported that they did not feel confident using technologies (*n* = 8) and/or had not previously used technology for any specific healthcare purposes (*n* = 12). Those who had used technology for healthcare purposes described using the English National Health Service (NHS) app (*n* = 11), GP websites to book appointments (*n* = 3), wearables (e.g. glucose monitors) prescribed by their doctor (*n* = 2), or a video call for a health consultation (*n* = 2).

**Table 1:** Summary of CLEARS groups represented in the participant sample

CLEARS domain	Frequency	Subcategory	Frequency
Culture	38	Diverse ethnicity (not White British)	14
		English as a second language	4
		Have a religious belief	20
Limiting conditions	11	Sight impairment	2
		Hearing impairment	4
		Both	5
Education	12	GSCE level	3
		Below GCSE	9
Age	12	61–70	8
		71–80	4
Residence	19	Rural area	0
		Deprived area	12
		No fixed address (lived in a sheltered accommodation)	7
Socioeconomic status	24	Retired	2
		Household annual income below £20,000	22

**Table 2:** Participant demographics

Participant ID, CLEARS domains represented	Data collection method	Gender	Ethnicity	Language	Religion	Limiting condition	Educational attainment	Age	Residence IMD^a^ score: 3 or below= deprived	Socio-economic status Household annual income below £20,000
9, ERS	Interview	Female	White	English	N/A	N/A	GCSE (or equivalent)	41-50	1	Yes
10, EARS	Interview	Female	White	English	N/A	N/A	GCSE (or equivalent)	61-70	3	Yes
11, LEAS	Interview	Female	White	English	N/A	Wears a hearing aid and has alternating vision	No qualifications	61-70	Postcode not provided	Yes
12, CEARS	Dual interview	Male	White	English	Greek Orthodox	N/A	No qualifications	61-70	3	Yes
13, CEARS	Dual interview	Female	White	Greek (fluent in English)	Greek orthodox	N/A	No qualifications	61-70	3	Yes
14, CA	Interview	Female	Asian / Asian British	English	Hindu	N/A	Postgraduate	61-70	10	Preferred not to say
15, CARS	Interview	Male	Mixed / Multiple ethnic groups	English	Christian (Catholic, Protestant or any other Christian denominations)	N/A	Postgraduate degree	61-70	1	Yes
16, CES	Focus group	Female	Asian / Asian British	English	Hindu	N/A	Level 2 English as 2nd language	51-60	4	Yes
17, CLERS	Focus group	Female	Asian / Asian British	English	Muslim	Visual impairment	No qualifications	31-40	Sheltered accommodation	Yes
18, C	Focus group	Female	Asian / Asian British	English	Muslim	N/A	First degree	31-40	6	No
19, CLRS	Focus group	Female	Asian / Asian British	English	Hindu	Hearing impairment	Postgraduate degree	31-40	1	Yes
20, CRS	Focus group	Female	Asian / Asian British	English	Muslim	N/A	First degree	21-30	Sheltered accommodation	Yes
21, CERS	Focus group	Female	Asian / Asian British	English	Muslim	N/A	Level 2 English as 2nd language	31-40	Sheltered accommodation	Yes
22, CR	Interview	Female	Arbab	Arabic	Muslim	N/A	First degree	41-50	2	No
23, CERS	Focus group	Female	Black / African / Caribbean / Black British	English	Christian (Catholic, Protestant or any other Christian denominations)	N/A	GCSE (or equivalent)	21-30	Sheltered accommodation	Yes
24, CRS	Focus group	Female	Asian / Asian British	English	Muslim	N/A	Postgraduate degree	31-40	Sheltered accommodation (Newcastle)	Yes
25, CRS	Focus group	Female	Asian / Asian British	English	Muslim	N/A	Postgraduate degree	21-30	Sheltered ccommodation	Yes
26, CLR	Focus group	Female	Iranian	Farsi (interpreter supported)	N/A	Both hearing and sight impairment	Prefer not to say	31-40	1	No
27, CERS	Focus group	Female	Taiwanese	English	Buddhist	N/A	No qualifications	31-40	Sheltered accommodation	Yes
28, AS	Interview	Male	White	English	N/A	N/A	A level (or equivalent)	61-70	9	Yes
29, CLEAS	Focus group	Male	White	English	Christian (Catholic, Protestant or any other Christian denominations)	Slight hearing loss	GCSE (or equivalent)	71-80	8	Yes
30, CLS	Focus group	Male	White	English	Christian (Catholic, Protestant or any other Christian denominations)	Yes	First degree	71-80	9	Yes
31, CA	Focus group	Male	White	English	Christian (Catholic, Protestant or any other Christian denominations)	N/A	Postgraduate degree o	71-80	4	Preferred not to say
32, LRS	Interview	Male	White	English	N/A	Visual impairment	A level (or equivalent)	41-50	3	Yes
33, ERS	Interview	Male	White	English	N/A	N/A	Level 1 maths and English	31-40	1	Yes
34, CLA	Focus group	Female	White	English	Christian (Catholic, Protestant or any other Christian denominations)	Deaf in one ear following neurosurgery operation	Postgraduate degree	61-70	10	No
35, LARS	Focus group	Female	White	English	N/A	Severe hearing loss	Postgraduate degree	71-80	3	No
36, CLRS	Focus group	Female	White	English	Christian (Catholic, Protestant or any other Christian denominations)	Deaf and visual impairment	Postgraduate degree	51-60	3	Yes
37, CLS	Focus group	Male	White	British sign language (interpreter supported)	N/A	Profoundly Deaf and short sighted	A level (or equivalent)	21-30	4	Yes

^a^IMD: Index of Multiple Deprivation.

### Key findings

**Fig. 1:**
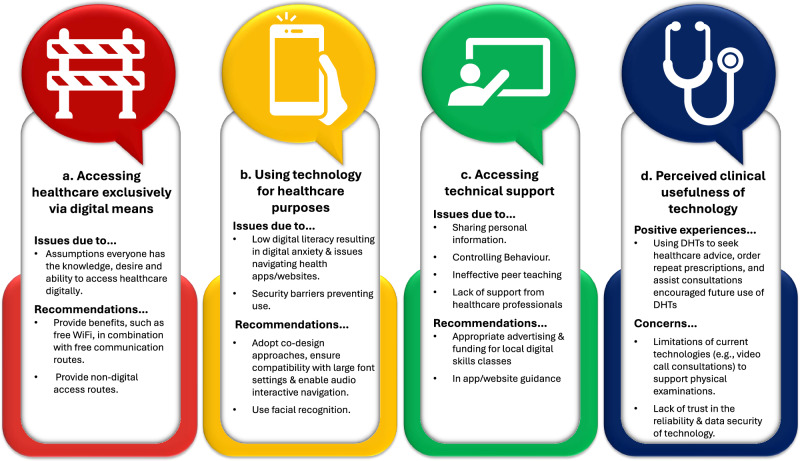
Summary of themes and sub-themes. **a** The first theme regarding accessing healthcare exclusively via digital means. **b** Second theme around the use of technology for healthcare purposes. **c** Third theme regarding accessing technical support. **d** The last theme discussing perceived clinical usefulness of technology within healthcare settings (DHTs digital health technologies).

Four key themes emerged around^1^ accessing healthcare exclusively via digital means^2^, using technology for healthcare purposes^3^, accessing technical support, and^4^ perspectives on the clinical usefulness of the technology (Table 3, Fig. 1). These themes are interlinked, for example, an individual with low digital literacy skills who struggles to use technology for healthcare purposes will likely experience barriers accessing healthcare exclusively via digital means. To ensure we present well-defined clear themes, key themes and subthemes are described in turn below using direct quotations. Participants interviewed in the piloting of the topic guide were assigned an identification number chronologically^1–8^, and their data was not included in the analysis. Participants in the main study were assigned an identification number^9–37^ followed by the letter(s) of the CLEARS group they represented (e.g., P9 ERS = participant who was interviewed ninth overall, has a low **E**ducational attainment, lived in a **R**esidency at risk of digital exclusion (e.g. deprived area), and has a low **S**ocioeconomic status).

**Table 3:** Description of the key themes and subthemes

Theme	Subtheme	Description
Accessing healthcare exclusively via digital means	Barriers to digital access routes	Barriers that occurred when accessing healthcare that was only available via digital means e.g., appointments could only be booked via a phone call, or upcoming appointments could only be confirmed via a weblink.
	Perceived solutions to improve access to healthcare	Providing resources to those who need support accessing devices and connectivity, and the need to provide non-digital access routes.
Using technology for healthcare purposes	Low digital literacy skills	A lack of understanding on how to use technology and difficulties when navigating health apps and websites.
	Security barriers	Aspects of authentication during the registration and login process of health apps that caused issues.
Accessing technical support	Overreliance on peer support	Issues that occur when an individual relies on support from their family or friends to aid the use of technology for healthcare purposes.
	Professional support services	Key areas limiting the scalability of technical support services within communities.
Perceived clinical usefulness of technology	Positive experiences	Positive experiences of using DHTs enhanced perceived usefulness of technology.
	Perceived limitations and lack of trust in digital technologies	Reflections on the limitations of current technology within healthcare and data security concerns.

### Accessing healthcare exclusively via digital means

Most participants experienced situations where healthcare providers had assumed that they had the required knowledge, desire and/or ability to access healthcare exclusively via digital means. One participant described an example where they were required to send a photograph of a wound to a healthcare professional during a remote consultation and explained how:

*“I automatically said, “Well, so for you to help me, I’ve got to go out and buy a camera, then I’ve got to take a photo and then I’ve got to go and get that photo developed then I’ve got to post it to you, is that what you’re saying?” and he [healthcare professional] said, “Well just send me a photo online.” […] [but] I do not have any form of the internet, no communication with the internet, no smartphone.” (P11 LEAS)*.

Other participants described receiving an automated no-reply SMS message containing a weblink that they needed to click on to confirm their upcoming hospital appointment. They reflected on how weblinks required access to the internet and they were not given an alternative way of confirming. Some participants decided to attend their local GP surgery in person to arrange an appointment but were told by the receptionist that appointments could only be made by telephone or via the online GP appointment system. Such experiences left some feeling like they were *“being forced into having the internet”*(P11 LEAS) in order to access healthcare; however, these participants acknowledged that they didn’t have the confidence to use it. Participants who identified as hard of hearing also described situations where appointments at the local GP surgery were only available to book via telephone, and they were unable to do this independently due to their hearing difficulties. Other participants described how the telephone lines were often very busy when trying to book a GP appointment, and they could be cut off prematurely due to poor signal and/or lack of minutes on their phone plan.

Many participants who experienced digital exclusion believed that healthcare providers should be able to provide free Wi-Fi *“so it’s possible to do the medical things online”* (P29 CLEAS). One participant proposed recording an individual’s ability to access digital healthcare services on their electronic healthcare record, so that “*when it comes up with your name and your date of birth [on the healthcare professionals’ screen] […] they [healthcare professional could] just put on their screens, ‘no internet connection’*” (P11 LEARS) to help identify and/or remind themselves of who might need alternative options. Most participants felt strongly that non-digital access routes to healthcare should always be provided, with one highlighting how *“if it all goes technology, I don’t know how I will cope*” (P34 CLA).

### Using technology for healthcare purposes

Many culturally diverse individuals and older adults were not motivated to use technology for healthcare purposes and felt overwhelmed when trying to learn how to use the technology; “[the] *internet, it’s like fireworks are coming inwards to my brain because I don’t understand it”* (P11 LEAS). This participant also felt that their low digital literacy ability was “*down to a lack of education and understanding, […] I don’t think I have [the] mental capability to learn about the internet*” (P11 LEAS). Those with more confidence using websites and apps described the challenges they had faced navigating these sites, with one participant explaining: “*what you’re trying to find out [booking an appointment], doesn’t come up on the list [drop down menu] […] you don’t know which one to press. Then you normally press the wrong one and after half an hour, you’ve got to start from scratch again*” (P31 CA). One participant suggested that *“more people from the community”* should come together with *“some of these technology experts”* (P35 LAR) to design and develop technologies that are more inclusive by identifying what needs to be changed to support a diverse range of users. Others also recommended the implementation of user guides within these apps and websites, including *“BSL [British Sign Language] videos on the [NHS and Local GP] website on how to book an appointment”* (P37 CLS).

Participants who had low educational attainment, lived in a deprived area or sheltered accommodation, and/or was on a low socioeconomic income, described various security barriers when using online health accounts. They explained how they needed photographic identification (e.g., passport) to register for the NHS app. This created a barrier for some who did not have a passport in their possession because it “ *is with [the] Home Office. I’m an asylum seeker*” (P21 CERS) or for those who could not afford to get one. Some participants without photographic identification found it difficult to upload a suitable picture, explaining how the instructions were unclear. Those who did register on the NHS app often reported difficulty remembering their passwords, with one participant explaining how they had *“to write stuff down. Well, when you write stuff down, you have a security issue*” (P15 CARS). One participant described how a family member had set up facial recognition for them and how this was a much easier alternative for “*anybody who wasn’t good at remembering them [passwords]*” (P10 EARS).

### Accessing technical support

Many participants turned to their family, friends or other individuals in their communities for support using technologies for healthcare purposes. One participant, who needed assistance from their neighbour to send an image to her healthcare professional, worried about sharing her private information with them; they also did not know who else to ask. Participants, who did receive one-to-one training from a family member or friend on how to use a device, often struggled with the technical terminology used as they did not understand it.

Participants who had experienced controlling relationships mentioned how their partners would forbid them from accessing technical support and/or attending digital skills classes at their local community centre. Other participants, who had attended these classes, found them useful. However, many had only heard about these classes through family or friends who had previously attended. One participant felt it was important to advertise these technical support services through various public places such as, *“doctor’s surgeries and libraries, bus stops, […] or schools”* and places of worship as *“you get massive communities there”* (P14 CA). Some participants perceived the need for more government funding for these services “*to move forward [to reach more people] very quickly*” (P35 LAR).

### Perceived clinical usefulness of technology within healthcare

Some participants described their positive experiences of using DHTs and how these experiences had encouraged them to continue using technologies for healthcare purposes. For example, participants valued being able to access healthcare information at any time via the NHS website and managing their prescriptions on the NHS app. One participant described how the NHS app presented previous prescription order dates and allowed them to change where they could collect their prescriptions. Another participant, in whom English was a second language, recalled how they showed their doctor the list of medications recorded on the NHS app as it was easier than verbally describing them in English.

*“I feel embarrassed because, “Oh, why don’t I remember? Is it because of the language [barrier]?” And it makes me feel stupid sort of thing. […] [When its] all there and the doctor could just read it; it’s a lot better” (P13 CEARS)*.

Another participant however questioned the accuracy of the information contained on the NHS app, explaining how the date and time of her upcoming hospital appointment was incorrect: *“it say 20*th *the something I’ve got appointment. I don’t understand why [it was different to the appointment date given to her by the healthcare professional]. So sometimes the technology doesn’t work for the purpose it has been designed [for]”* (P14 CA). Many participants highlighted how digital consultations (e.g., video) should not replace some in-person consultations, as there was a perceived need for the latter to discuss certain ailments. For example, one participant described how:

*“I told her [a GP] all about it [her neck/ throat pain], she did initially say to me I think it’s caused by the muscles […] she then had a good feel around. And then she agreed with what she [initially] said, […] its muscle pain.[…] I felt much more reassured. But if that had been video she wouldn’t have been able to feel there and I wouldn’t have felt the service was as good” (P36 CLRS)*.

Some participants also raised concerns regarding the safety and security of their health data on DHTs as they *“wouldn’t want anyone to know what medication you’re [they were] on or what treatment you’ve [they] had, or what operations you’ve [they] had […] you wouldn’t want that to leak”* (P9 ERS). Other participants were concerned around medical data being contained on their digital device: *“I don’t want any medical data sort of on me phone, I could lose the phone”* (P32 CLRS).

## Discussion

This study explored underserved groups’ perspectives and experiences of DHTs, and how they would like to be supported in the future. Our findings described the barriers underserved groups experienced when trying to access healthcare exclusively via digital means, the challenges of trying to use technology for healthcare purposes, issues accessing technical support, and perceptions on the clinical usefulness of technologies within healthcare. Participants made various recommendations to help support digital inclusion, such as healthcare professionals providing resources (e.g., free devices) to those who needed support, as well as alternative non-digital options especially when physical examinations are needed, and co-designing technology to improve usability. To further assist the use of DHTs, some participants suggested implementing in-app/website user-guides, and increasing funding for technical support services via community centres. These findings highlight various ways to support digital health equity by identifying any issues raised across intersecting demographic groups.

Various voluntary, community and social enterprise (VCSE) organisations do provide resources to aide digital inclusion in the UK. For example, The Good Things Foundation (a digital inclusion charity) have developed a National Device Bank and Databank that provides pre-used devices to the UK adult population who have insufficient access, alongside a free pre-paid SIM card with mobile data^25,26^. The NHS have recently published an inclusive digital healthcare framework, which advocates for the collaboration between the NHS and VCSE organisations to support the provision of free devices and data to those patients who need it^27^. This has supported collaborations between The South Tees Hospitals NHS Foundation Trust maternity service in the North East of England and The Good Things Foundation to provide patients who are at risk of digital exclusion with pre-used devices and pre-paid data plans^28^. However, further evaluation is needed in terms of scalability or effectiveness of these interventions (e.g., how many patients availed of this digital support and whether it improved their experiences and/or access of maternity services) in order to demonstrate the impact and benefit(s) of these services. To support their scalability and reach, the UK government’s latest ‘Digital Inclusion Action Plan’ published in 2025 highlights how support may be provided to VCSE organisations delivering educational digital skills services and the development of inclusive learning (e.g., translators and interpreters) as part of the new Digital Inclusion Innovation Fund^29^. This fund has been designed to support local initiatives to meet the specific and the diverse digital-related needs of local communities^29^.

The findings of our study also highlighted the need for non-digital options to be provided to access healthcare services, thus supporting those who lack confidence in using DHTs, have poor digital literacy skills, and/or prefer face-to-face communication. The NHS has recognised the importance of delivering both digital and non-digital options to balance the needs of those who are digitally excluded in an inclusive framework released in 2023^27^. Various UK policy documents produced by organisations such as Healthwatch (a UK based organisation which voices the concerns and experiences of healthcare users to improve healthcare services)^30^, and the Kings Fund (an independent think tank working to improve health and care in England) have also highlighted the importance of providing both options^31^. However, NHS England released plans in 2025 to set up an ‘online hospital’ which will not have a physical site and will only connect patients with healthcare professionals via digital means^32^. Further work is needed to evaluate the feasibility and economic impact of implementing and maintaining two access routes (digital and non-digital).

Many underserved groups in this qualitative study described how they found it hard to navigate certain health-related apps (e.g., the NHS app) and/or websites (e.g., GP practise websites), and were prevented from registering for an online account by certain security features. The UK government have promoted ‘secure by design principles’ which are mandatory for Government departments and require security processes of digital public services (including digital healthcare and DHTs) to be fit for purpose^33^. There are also UK standards in place which mandate that public-facing websites and software be *‘perceivable, operable, understandable and robust’* to ensure that those with visual and hearing impairments, low reading ability (reading age of 9) and/or those who are not fluent in English can access and understand the information provided^34^. The UK Equality Act 2010 states that healthcare services cannot directly discriminate against those who cannot afford photographic identification and/or those who are seeking asylum, many of whom may not be fluent in English^35^. The wider literature suggests that technical security developers often prioritise mitigating cybersecurity attacks and other breaches of data, without acknowledging how this might hinder a user’s ability to operate the resulting system^36,37^. Additionally, little research has been conducted exploring the needs of individuals from underserved groups to help identify inclusive user-friendly features. For example, Zhou et al. conducted a study exploring user-friendly security features in health-related apps that also reduced security and privacy concerns amongst users^38^. They found 43.6% (n = 51) of participants felt regular password updates would help^39^. However, this study only included residents in Greater Pittsburgh (Pennsylvania, US) who were native English speakers, well educated, and had at least 3 years of experience in using a smartphone before the study^38^. Our study also highlighted how underserved groups experiencing digital exclusion often struggled to recall their password, with many engaging in unsafe practices such as writing down their passwords. The use of biometrics (e.g., facial recognition) was suggested to overcome issues regarding password recall; however, this would exclude those who do not have access to technology with such capabilities (e.g. a smartphone with a camera). There are also growing concerns relating to the use of biometric algorithms, with reports of lower accuracy in facial recognition software for East Asian faces compared to Caucasian faces; this may be due to a lack of diversity in the data when training algorithms^40^.

Seeking technical support to aid the use of DHTs was a common strategy used by underserved groups in our study. However, many felt uncomfortable doing so and/or could experience controlling behaviours from a family member. Digital technologies can be used to threaten, harass, control, and/or punish an individual (technology-facilitated domestic abuse)^41^, and can further exacerbate digital exclusion within healthcare as it removes an individual’s independence, choice and/or control^42^. However, very little research has been conducted to explore the unique barriers domestic abuse victims have experienced regarding digital exclusion within healthcare, and different types of appropriate and effective digital inclusion strategies. Some participants from under-served groups in our study and the Good Things Foundation^43^ have suggested DHT developers create user guides (e.g., printable guides, video tutorials) that are available in different languages within health-related apps and/or websites to support an individual to use a DHT without assistance from others^43^. Technical support provided by local community centres was also perceived by individuals from underserved groups to help reduce digital anxiety, increase confidence and improve digital literacy skills. This support could take the form of one-to-one or in small group sessions that are tailored to an individual’s needs and interests (i.e., the specific type of technology that they want to use and what they want to use it for). It could be conducted in an informal manner (e.g., without the use of any formal assessments) to support those with poor past experiences of educational systems and/or a lack of confidence to learn how to use technologies^44^. Appropriate pictures, emojis and/or icons should also be used to facilitate communication and learning amongst those whose first language is not English^45^. Venues that host support services must also provide a suitable environment (i.e., offer free Wi-Fi, free car parking and/or good public transport links)^46^. Study participants called for more UK government funding to increase the awareness of available support services and the nuances of different social contexts for vulnerable individuals from under-served groups^47–49^. For example, homeless women are less likely to attend homeless shelters when compared to men, as they may have previously been victimised by men and/or do not feel comfortable in a shelter with men^47^. Therefore, homeless shelters may not be the most appropriate place to advertise support services for homeless women, and alternatives such as domestic abuse shelters should be considered to reach this population. The content of these advertisements (e.g., framing of the message and imagery) must also be carefully considered. It should contain real-life examples of the benefits of DHTs that resonate with the community so as to help motivate people to engage with the services^50^.

Our study used multiple strategies to ensure rigour and trustworthiness of our findings. The topic guide was piloted with individuals who represent the target population, data was collected until data saturation was reached, and various contextual and societal factors were acknowledged (e.g., intersectionality, and regional health and digital inequities). This important contextual information has been omitted from previous studies exploring the implementation of new interventions or strategies to promote digital equity in healthcare^51,52^. However, it must be noted that inter-coder reliability tests were not performed during the analysis. A further strength of this study is the use of a unique inclusive recruitment approach to obtain a wide range of participants from different intersecting underserved groups and a breadth of different opinions, perspectives and experiences to help provide insight into how we might support digital health equity for everyone. However, all participants were from the North East and may not reflect the views of those from other geographic regions. Despite our best efforts, we were unable to recruit any participants from rural areas (due to transport issues) and only three non-English speaking individuals participated (due to limited access to multilingual translators). This may limit the transferability of our findings to those living in remote geographical areas and individuals who do not speak English. Thus, future research must explore the experiences and perspectives of those from other geographic or cultural contexts. Participants voluntarily consented to take part in this study; there was the risk of self-selection bias as those who participated may have been more motivated and able to take part. However, we believe our extensive recruitment approach sought to minimise self-selection bias to some extent. One participant who took part in a focus group indicated on the expression of interest form that they only represented one CLEARS group, (Cultural factors: ethnicity) which did not meet the eligibility criteria (i.e., self-identify with two or more groups). However, the organisation that she was recruited through indicated that she may have been a victim of domestic abuse and may have preferred not to disclose personal information. Thus, her inclusion helped provide valuable insights in an area where there has been little to no research conducted exploring strategies to support digital inclusion within healthcare for victims of domestic abuse. Although these findings help us understand the issues experienced by different underserved groups at risk of digital exclusion, many participants identified with four socio-demographic groups; consequently, it would be very difficult (if not impossible) to separate out individual demographic groups due to this intersectionality. This study highlights the issues that arise across these CLEARS groups and where resources should directed to meet their needs.

To conclude, this qualitative study identified four key themes describing the barriers experienced by underserved groups living in North East of England when trying to access or use DHTs, as well as their thoughts on how to improve digital inclusion. This information can be used by healthcare professionals and DHT developers to help improve digital inclusion, as well as factors that need to be considered when designing, developing and implementing new DHTs. Future research is needed to explore stakeholders’ perspectives on the feasibility of implementing various recommendations raised in this study.

## Methods

### Participants

Participants were recruited across the North East of England; 28% of residents in this region have low digital competency^53^, and a higher percentage (5%) do not use internet enabled devices, internet-based communication routes, or internet information sources (when compared to England’s average of 3.6%)^53^. We used an inclusive recruitment approach to target residents currently living in the North East who have experienced digital exclusion. This involved a combination of purposeful snowball sampling^54^, and in-person recruitment with local voluntary, community, and social enterprise organisations. We also engaged with the local council who currently provide services to support those at risk of digital exclusion, such as digital skills classes. Individuals interested in participating were asked to complete an expression of interest form to assess eligibility and monitor diversity. Eligible participants were contacted after the recruitment session via their preferred mode of contact (e.g., phone call, text, email etc.) to invite them to participate.

We conducted a scoping review of the literature to identify socio-demographic factors that could put an individual at risk of digital exclusion. We developed and published the CLEARS framework^21,39^, which encompasses these socio-demographic factors (**C**ultural factors: ethnicity, language and religion, **L**imiting conditions: visual and hearing impairments, low **E**ducational attainment: government mandated level or below, older **A**ge: over 65 years old, place of **R**esidence: rural or deprived areas and/or those without a fixed address, and low **S**ocioeconomic status: low income and/or unemployed individuals)^21,39^. The CLEARS framework acknowledges the intersectionality of these six different socio-demographic factors within society, and how this places an individual at greater risk of digital exclusion^21,39^. To be included in this study, participants needed to self-identify with at least two of the factors within the CLEARS framework to ensure that this intersectionality was represented in our sample. Participants also needed to be over 18 years old and have self-reported as having limited access to a list of common household technologies, such as smartphones, mobile phones, laptops etc. (≤3 out of 10), and/or used these technologies for limited purposes (≤2 out of 7 common uses listed, such as for work, study, entertainment, socialising, etc.).

### Procedure and data collection

Ethical approval was granted by the Newcastle University Faculty of Medical Science Ethics Committee (2617_2/35084) and was conducted in accordance with the principles of the Declaration of Helsinki^55^.

Expression of Interest forms were completed during recruitment and before participation. The form contained 15 questions to gather demographic data (preferred gender, ethnicity, preferred spoken language, religion, hearing and/or visual impairments, highest educational attainment, age, annual income range (above or below the UK government level of low income (£20,000)), and postcode), everyday access and use of common household technologies, confidence using technologies, and contact details (Supplementary Information 1).

Verbal and written informed consent were obtained before participation. Participants chose to take part in either a semi-structured interview or focus group at a mutually convenient time and place. These interviews and focus groups explored participants’ experiences of DHTs, and their opinions and perspectives on different strategies to support digital inclusion. A flexible topic guide was used during all interviews and focus groups. The topic guide was informed by the findings of our systematic review which identified key strategies to support digital inclusion^21^, and was piloted with eight individuals who represented at least three of the CLEARS factors (Supplementary Information 2). All focus groups and interviews were conducted in-person at a suitable location, such as the community centre or Newcastle University, between 25th November 2023 and 11th April 2024. All focus groups and 10 interviews were conducted by a female qualitative researcher (SW) who at the time of this study was a PhD student and Research Assistant with considerable experience of conducting research with vulnerable groups. One interview was conducted by a female native Arabic speaker (NH) who was a Research Associate with PhD in public health. The researchers did not have any prior relationship with participants, nor did they disclose their background (other than being a researcher) to participants. Appropriate assistance to facilitate the focus groups or interviews (e.g., a speech-to-text operator, British Sign Language translator etc.) were also provided, when needed. Focus groups and interviews were audio recorded with permission, transcribed verbatim by a transcription company (University Transcriptions), anonymised and checked by the research team for accuracy. Field notes were taken throughout the data collection process and were used to provide additional context for the analysis and support the authors’ critical reflection. Data collection was carried out until data saturation was reached (i.e., when no new insights or themes emerged for data collection). This was done by assessing the duration and depth of discussion within the interviews and focus groups and the occurrence of themes and concepts in the data during research team meetings^56^.

Participants completed a follow-up questionnaire (physical paper copies were provided) straight after participating in the focus group or interview. This questionnaire contained three multiple choice questions to gather information on their experience of taking part in this study and their use of technology specifically for health-related purposes (see Supplementary Information 3). Participants were remunerated with a £20 supermarket voucher after completion of the questionnaire.

### Data analysis

Transcripts from the focus groups and interviews were uploaded to N-Vivo (QSR, version 14.23.2) and analysed using an inductive reflexive thematic analysis approach^57^. The six key phases of thematic analysis were followed^58,59^, starting with the lead author (SW) reading and re-reading the transcripts. Initial codes were then generated without any pre-conceived theories or concepts using the ‘OSOP’ (‘one sheet of paper’) technique^60^. Preliminary themes were generated by reflecting on the notes generated using the OSOP technique and re-reading the transcripts to identify deeper connections between the descriptive codes and significant broader patterns of meaning. The researchers also investigated any issues across intersecting demographic groups and any suggestions on how they should be addressed. As our participants identified with multiple socio-demographic factors that could put them at risk of digital exclusion, we could not sufficiently compare one demographic group against another. Themes were further refined by mapping them visually to identify connections between themes. Such visuals aided discussions with the research team (CT, SPS, and RMA), in which quotes and themes were discussed to consider variances in interpretation and support the refinement and consensus of key themes. The research team were experienced qualitative researchers at various career stages, with academic backgrounds in digital health equity, pharmacy and neuroscience. This multidisciplinary approach mitigated bias from each researcher’s own interests and ensured a comprehensive understanding of participants’ experiences. The final themes and codes were then applied systematically across the whole data set.

Quantitative data gathered via the expression of interest form and follow-up questionnaire was described using the frequencies of different metrics (e.g., ethnicities). Postcode information indicated deprivation through the national datasets like the Index of Multiple Deprivation which ranks small areas based on factors like income, employment, education, and crime^61^.

### Rigour and trustworthiness

Rigour was upheld by following the Standards for Reporting Qualitative Research (SRQR)^62^, and completing the Consolidated criteria for reporting qualitative research (COREQ) checklist (Supplementary Information 4)^63^. Trustworthiness was established by ensuring credibility, transferability, dependability, and confirmability^64^. Credibility was addressed by piloting the topic guide to ensure the questions could be easily understood by participants, and data was collected using different methods reducing the impact of potential bias from a single data source^65^. Transferability was addressed by providing a rich description of various aspects of this research (e.g., study setting, recruitment strategy, eligibility criteria, study procedures) to allow readers to judge the transferability of the research findings^65,66^. Dependability was achieved by creating a comprehensive log of decisions, rationales, raw data, field notes, transcripts and reflections on daily logistics to ensure the research process was clearly documented^66,67^. To establish confirmability, peer debriefing was conducted with all authors throughout the study to review decisions, interpretations, conclusions, and provide an external check on the research process^67^.

## Supplementary information

Supplementary info 1-4

## Data Availability

The data sets generated and analysed during this study are not publicly available due to a potential breach of participant confidentiality. Anonymized data sets may be available from the corresponding author on reasonable request.

## References

[1] CEDEFOP. Digital Exclusion. https://www.cedefop.europa.eu/en/tools/vet-glossary/glossary/digitale-uitsluiting (2023).

[2] Audit Scotland. Tackling digital exclusion. https://audit.scot/publications/tackling-digital-exclusion (2024).

[3] Data Rich. Digital Exclusion https://www.datarich.info/digital-exclusion/.

[4] UK Parliament House of Lord Communications and Digital Committee. Digital exclusion https://committees.parliament.uk/publications/40662/documents/198365/default/ (2023).

[5] ITU. Facts and Figures 2021: 2.9 billion people still offline https://www.itu.int/hub/2021/11/facts-and-figures-2021-2-9-billion-people-still-offline/ (2021).

[6] Lloyds Bank. Essential Digital Skills Report https://charnwood.moderngov.co.uk/documents/s9362/DTSP%2029%20March%202022%20-%20Itm%20XX%20-%20Ann%203%20-%20211109-lloyds-essential-digital-skills-report-2021.pdf (2021).

[7] Cebr. The economic impact of digital inclusion in the UK. https://www.goodthingsfoundation.org/policy-and-research/research-and-evidence/research-2024/digital-inclusion-uk-economic-impact?gad_source=1&gad_campaignid=22623478783&gbraid=0AAAAAqUqZ8nUDJLKoIEJMfLSB6Huiym5L&gclid=Cj0KCQjwqebEBhD9ARIsAFZMbfyOQf5XZ_l9 (2022).

[8] Lopez Perales, C. R. et al.Mobile health applications for the detection of atrial fibrillation: a systematic review. *EP Europace* **23**, 11–28 (2021). 10.1093/europace/euaa139 PMC7842109 33043358

[9] National Institute for Health and Clinical Excellence. Evidence standards framework for digital health technologies https://www.nice.org.uk/corporate/ecd7/resources/evidence-standards-framework-for-digital-health-technologies-pdf-1124017447605.

[10] Arora, S., Venkataraman, V., Zhan, A., Donohue, S., Biglan, K. M., Dorsey, E. R. & Little, M. A. Detecting and monitoring the symptoms of Parkinson’s disease using smartphones: A pilot study. *Parkinsonism Relat. Disord.* **21**, 650–653 (2015). 25819808 10.1016/j.parkreldis.2015.02.026

[11] Yao, R. et al. Inequities in health care services caused by the adoption of digital health technologies: scoping review. *J. Med. Internet Res.* **24**, e34144 (2022). 35311682 10.2196/34144 PMC8981004

[12] NHS England. Analogue to Digital. https://www.england.nhs.uk/north-east-yorkshire/our-work/10-year-health-plan/analogue-to-digital/.

[13] NHS. Remote ECG monitoring to support mental health patients in the North East and Yorkshire. NHS England. https://transform.england.nhs.uk/covid-19-response/technology-nhs/remote-ecg-monitoring-to-support-mental-health-patients-in-the-north-east-and-yorkshire/#:~:text=The%20project%20aims%20to%3A,process%20quicker%20for%20the%20patient.

[14] Honeyman, M., Maguire, D., Evans, H. & Davies, A. Digital technology and health inequalities: a scoping review. Cardiff: Public Health Wales NHS Trust. Digital technology and health inequalities: a scoping review Cardiff: Public Health Wales NHS Trust. 2020.

[15] Parson, L. Considering positionality: The ethics of conducting research with marginalized groups. In *Research methods for social justice and equity in education.* 15–32 (Springer International Publishing, Cham, 2019).

[16] Wallerstein, N. et al. Power dynamics in community-based participatory research: A multiple–case study analysis of partnering contexts, histories, and practices. *Health Educ. Behav.* **46**, 19S–32S (2019). 31549557 10.1177/1090198119852998

[17] World Health Organization. Health inequities and their causes. https://www.who.int/news-room/facts-in-pictures/detail/health-inequities-and-their-causes (2018).

[18] Improving inclusion of under-served groups in clinical research: Guidance from include project. NIHR. https://www.nihr.ac.uk/documents/improving-inclusion-of-under-served-groups-in-clinical-research-guidance-from-include-project/25435 (January 2022 v2.0).

[19] Richardson, S., Lawrence, K., Schoenthaler, A. M. & Mann, D. A framework for digital health equity. *npj Digit. Med.* **5**, 119, 10.1038/s41746-022-00663-0 (2022). 35982146 10.1038/s41746-022-00663-0 PMC9387425

[20] Gleason, K. & Suen, J. J. Going beyond affordability for digital equity: Closing the “Digital Divide” through outreach and training programs for older adults. *J. Am. Geriatrics Soc.* **70**, 75–77, 10.1111/jgs.17511 (2022). 10.1111/jgs.17511 PMC8742748 34648663

[21] Wilson, S. et al. Recommendations to advance digital health equity: a systematic review of qualitative studies. *NPJ Digital Med.* **7**, 173 (2024). 10.1038/s41746-024-01177-7 PMC11217442 38951666

[22] Wilson-Menzfeld, G. et al. Understanding Digital Exclusion across North Tyneside (published February 2023). Available from: https://researchportal.northumbria.ac.uk/ws/portalfiles/portal/92647654/Final_report_Understanding_Digital_Exclusion_across_North_Tyneside.pdf.

[23] McCall, T., Asuzu, K., Oladele, C. R., Leung, T. I. & Wang, K. H. A socio-ecological approach to addressing digital redlining in the United States: a call to action for health equity. *Front. Digital Health* **4**, 897250 (2022). 10.3389/fdgth.2022.897250 PMC9339607 35924138

[24] Richardson, S., Lawrence, K., Schoenthaler, A. M. & Mann, D. A framework for digital health equity. *NPJ Digital Med.* **5**, 119 (2022). 10.1038/s41746-022-00663-0 PMC9387425 35982146

[25] Good Things Foundation. Our digital inclusion services. https://www.goodthingsfoundation.org/our-services.

[26] Good things foundation. What is the National Device Bank? https://www.goodthingsfoundation.org/our-services/national-device-bank.

[27] NHS England. Inclusive digital healthcare: a framework for NHS action on digital inclusion https://www.england.nhs.uk/long-read/inclusive-digital-healthcare-a-framework-for-nhs-action-on-digital-inclusion/ (2024).

[28] North East and North Cumbria Local Maternity and Neonatal System. South Tees midwife improves digital inclusion https://northernlms.org/south-tees-midwife-improves-digital-inclusion/.

[29] UK Government. Digital Inclusion Action Plan: First Steps (published: February 26th 2025). Available from: https://www.gov.uk/government/publications/digital-inclusion-action-plan-first-steps/digital-inclusion-action-plan-first-steps.

[30] Healthwatch. Digital health and Care: A report on local experiences in Suffolk and North East Essex https://healthwatchsuffolk.co.uk/wp-content/uploads/2021/05/Digital-Health-and-Care-Final-Copy-1.pdf (2021).

[31] Kings Fund. Policy brief: Ensuring digitally enabled health care is equitable and effective for all https://www.kingsfund.org.uk/insight-and-analysis/evidence-and-consultations/ensuring-digitally-enabled-health-care-equitable-effective-for-all (2023).

[32] NHS England. New NHS online hospital to give patients more control over their care https://www.england.nhs.uk/2025/09/new-nhs-online-hospital-to-give-patients-more-control-over-their-care/ (2025).

[33] Government Security Central Digital and Data Office (CDDO), Cabinet Office. Secure by Design Principles. https://www.security.gov.uk/policy-and-guidance/secure-by-design/principles/#4-design-usable-security-controls.

[34] Legislation.gov.uk. The Public Sector Bodies (Websites and Mobile Applications) (No. 2) Accessibility Regulations https://www.legislation.gov.uk/uksi/2018/952/contents (2018).

[35] UK Government. Equality Act 2010 https://www.legislation.gov.uk/ukpga/2010/15/contents (2010).

[36] Bures, V. Comparative analysis of system dynamics software packages. *Int. Rev. Model. Simul. (IREMOS)* **8**, 245–256 (2015).

[37] Al-Sarayreh, K. T., Hasan, L. A. & Almakadmeh, K. A trade-off model of software requirements for balancing between security and usability issues. *Int. Rev. Comput. Softw.* **10**, 1157–1168 (2015).

[38] Zhou, L., Bao, J., Watzlaf, V. & Parmanto, B. Barriers to and facilitators of the use of mobile health apps from a security perspective: mixed-methods study. *JMIR mHealth Health* **7**, e11223 (2019). 10.2196/11223 PMC6488955 30990458

[39] Wilson, S., Tolley, C., McArdle, R., Slight, R. & Slight, S. Who is most at risk of digital exclusion within healthcare? *Int. J. Pharm. Pract.* **32**, i3–i4 (2024).

[40] Phillips, P. J., Jiang, F., Narvekar, A., Ayyad, J. & O’Toole, A. J. An other-race effect for face recognition algorithms. *ACM Trans. Appl. Percept. (TAP)* **8**, 1–1 (2011).

[41] Henry, N., Vasil, S., Flynn, A., Kellard, K. & Mortreux, C. Technology-facilitated domestic violence against immigrant and refugee women: A qualitative study. *J. Interpers. Violence* **37**, NP12634–NP12660 (2022). 33719681 10.1177/08862605211001465

[42] Good Things Foundation. Mitigating risks of digital exclusion in health systems https://www.goodthingsfoundation.org/policy-and-research/research-and-evidence/research-2024/health-inequalities-digital-exclusion (2024).

[43] Good Things Foundation. Health inequalities and mitigating risks of digital exclusion (2nd edition) https://www.goodthingsfoundation.org/dam/jcr:43bc6a26-98ae-4304-bc4e-fcbcfa010d60/GoodThings_HealthInequalitiesAndMitigatingRisksOfDigitalExclusion_2024.pdf file:///C:/Users/wisar/Downloads/GoodThings_HealthInequalitiesAndMitigatingRisksOfDigitalExclusion_2024%20(3).pdf (2024).

[44] Good Things Foundation. Basic Digital Skills: Expert Overview https://www.goodthingsfoundation.org/policy-and-research/what-works-colab/basic-digital-skills-briefing#routes file:///C:/Users/wisar/Downloads/GoodThings_HealthInequalitiesAndMitigatingRisksOfDigitalExclusion_2024%20(3).pdf (2025).

[45] Good Things Foundation. Supporting digital inclusion for adults with low English language skills https://www.goodthingsfoundation.org/policy-and-research/research-and-evidence/research-2024/supporting-digital-inclusion-adults-with-low-english-language-skills file:///C:/Users/wisar/Downloads/GoodThings_HealthInequalitiesAndMitigatingRisksOfDigitalExclusion_2024%20(3).pdf (2020).

[46] Good Things Foundation. Digital Skills in rural settings using Learn My Way. https://www.goodthingsfoundation.org/discover/digital-inclusion-resources/learning-and-skills/using-learn-my-way-with-rural-learners file:///C:/Users/wisar/Downloads/GoodThings_HealthInequalitiesAndMitigatingRisksOfDigitalExclusion_2024%20(3).pdf.

[47] de Vet, R. et al. Differences between homeless women and men before and after the transition from shelter to community living: A longitudinal analysis. *Health Soc. Care Community* **27**(Sep), 1193–1203 (2019). 30989763 10.1111/hsc.12752 PMC6850267

[48] Springs Rescue Mission. A Hidden Side of Homelessness: Why Women Avoid Homeless Shelters https://www.springsrescuemission.org/articles/a-hidden-side-of-homelessness-why-women-avoid-homeless-shelters#:~:text=Homeless%20women%20avoid%20staying%20in%20shelters&text=Some%20studies%20have%20found%20that,in%20a%20shelter%20with%20men.

[49] Crystal, S. Homeless men and homeless women: The gender gap. *Urban Soc. change Rev.* **17**, 2–6 (1984).

[50] Age UK. Helping older people get online. Available from: https://www.ageuk.org.uk/our-impact/programmes/digital-skills/.

[51] Waters, E. et al. Essential components of public health evidence reviews: capturing intervention complexity, implementation, economics and equity. *J. public health* **33**(Sep), 462–465 (2011). 10.1093/pubmed/fdr064 21859880

[52] Pfadenhauer, L. M. et al. Making sense of complexity in context and implementation: the Context and Implementation of Complex Interventions (CICI) framework. *Implement. Sci.* **12**(Dec), 1–7 (2017). 28202031 10.1186/s13012-017-0552-5 PMC5312531

[53] LLYODS bank. 2023 Consumer Digital Index: The UK’s largest study of digital and financial lives. https://www.lloydsbank.com/assets/media/pdfs/banking_with_us/whats-happening/231122-lloyds-consumer-digital-index-2023-report.pdf (2023).

[54] Naderifar, M., Goli, H. & Ghaljaie, F. Snowball sampling: A purposeful method of sampling in qualitative research. *Strides Dev. Med. Educ. J.* **14**, 1–6 (2017).

[55] Bibbins-Domingo, K., Brubaker, L. & Curfman, G. The 2024 revision to the Declaration of Helsinki: modern ethics for medical research. *JAMA* **333**(Jan), 30–31 (2025). 39425945 10.1001/jama.2024.22530

[56] Malterud, K., Siersma, V. D. & Guassora, A. D. Sample size in qualitative interview studies: guided by information power. *Qualitative Health Res.* **26**(Nov), 1753–1760 (2016). 10.1177/1049732315617444 26613970

[57] Byrne, D. A worked example of Braun and Clarke’s approach to reflexive thematic analysis. *Qual. Quant.* **56**(Jun), 1391–1412 (2022).

[58] Cooper, H. E. Research designs: Quantitative, qualitative, neuropsychological, and biological. *Am. Psychol.* **2**, x–701 (2012).

[59] Braun, V. & Clarke, V. One size fits all? What counts as quality practice in (reflexive) thematic analysis? *Qualitative Res. Psychol.* **18**, 328–352 (2020).

[60] Ziebland, S. & McPherson, A. Making sense of qualitative data analysis: an introduction with illustrations from DIPEx (personal experiences of health and illness). *Med. Educ.* **40**, 405–414 (2006). 16635119 10.1111/j.1365-2929.2006.02467.x

[61] UK Government, Ministry of Housing, Communities & Local Government. English indices of deprivation https://imd-by-postcode.opendatacommunities.org/imd/2019 (2019).

[62] O’Brien, B. C., Harris, I. B., Beckman, T. J., Reed, D. A. & Cook, D. A. Standards for reporting qualitative research: a synthesis of recommendations. *Academic Med.* **89**, 1245–1251 (2014). 10.1097/ACM.0000000000000388 24979285

[63] Tong, A., Sainsbury, P. & Craig, J. Consolidated criteria for reporting qualitative research (COREQ): a 32-item checklist for interviews and focus groups. *Int. J. Qual. Health Care* **19**, 349–357 (2007). 17872937 10.1093/intqhc/mzm042

[64] Nowell, L. S., Norris, J. M., White, D. E. & Moules, N. J. Thematic analysis: Striving to meet the trustworthiness criteria. *Int. J. Qualitative Methods* **16**(Sep), 1609406917733847 (2017).

[65] Ahmed, S. K. The pillars of trustworthiness in qualitative research. *J. Med., Surg., Public Health* **2**, 100051 (2024).

[66] Lincoln, Y. S. & Guba, E. G. Naturalistic inquiry. Sage, Newberry Park; 1985.

[67] Halpren, E. S. Auditing naturalistic inquiries: The development and application of a model (Unpublished doctoral dissertation). Indiana University, Bloomington. (1983).

