# Supplementary material for: Under-served groups’ perspectives on mitigating digital exclusion within healthcare in the North East of England
Source: NPJ Digit Public Health. 2026 Jul 3;1(1):19. doi: 10.1038/s44482-026-00022-w (PMC13331730; doi:10.1038/s44482-026-00022-w)
Supplement: Supplementary file 1 — Supplementary info 1-4 [file 44482_2026_22_MOESM1_ESM.pdf]

Interview guide

I would like to start by thanking you for taking the time to talk to me today. I am interested in hearing your experiences of accessing your doctor and opinions on using technology to help you access them. Do you have any questions before we begin?

Before we start, is it ok if I record our conversation? Everything you say will be kept confidential.

1) Can you tell me about any challenges you have experienced when trying to access your doctor or any type of healthcare?

2) Have you ever used or heard of any technology, such as an app or video call, that could help you access your doctor or other health information?

3) Have you got any thoughts about how can we improve technology that is been used for a health purposes?

I've been reading about different ways to support people like yourself in accessing and using technology for health purposes, as well as helping people feel more motivated to use them.

4) The first way is about making the technology easy to use. So, what makes some technology easy for you to use?

5) The second is about being provided with equipment. What would you like to be provided with to help you access and use technology for a health purpose and why?

6) The last one is about technical support. Can you tell me if this is something you would like and what this support should look like?

7) Are there any other things you would like to see in place to help you use, access or feel more motivated to use technology for health purposes?

   - How would you like to hear about any new changes to support that is available?

Study ID: (filled in by research team)

Expression of interest form

1. What gender do you identify as?
   - ☐ Female (same as my sex assigned at birth)
   - ☐ Male (same as my sex assigned at birth)
   - ☐ Transgender female
   - ☐ Transgender male
   - ☐ Genderfluid
   - ☐ Non-binary
   - ☐ Other, please specify
   - ☐ Prefer not to say.

2. How old are you?
   - ☐ Under 18
   - ☐ 18- 20
   - ☐ 21-30
   - ☐ 31-40
   - ☐ 41-50
   - ☐ 51-60
   - ☐ 61-70
   - ☐ 71-80
   - ☐ 81-90
   - ☐ Over 91
   - ☐ Prefer not to say.

3. Please specify your ethnicity.
   - ☐ White
   - ☐ Mixed / Multiple ethnic groups
   - ☐ Asian / Asian British
   - ☐ Black / African / Caribbean / Black British
   - ☐ Other ethnic group, please specify
   - ☐ Prefer not to say.

4. What is your preferred language to communicate in?
   - ☐ English
   - ☐ British sign language
   - ☐ Arabic
   - ☐ Polish
   - ☐ Romanian

- ☐ Panjabi
- ☐ Urdu
- ☐ Other, please specify

  [ ]

- ☐ Prefer not to say.

5. Do you have a religious faith?

   - ☐ Christian (Catholic, Protestant or any other Christian denominations)
   - ☐ Hindu
   - ☐ Jewish
   - ☐ Muslim
   - ☐ Other, (please specify)

     [ ]

   - ☐ I am not religious
   - ☐ Prefer not to say.

6. What is your highest educational attainment?
   - ☐ No qualifications
   - ☐ GCSE (or equivalent)
   - ☐ A level (or equivalent)
   - ☐ First degree (e.g. BA, BSc)
   - ☐ Postgraduate degree or professional qualification (e.g., qualified teacher)
   - ☐ Other, please specify

     [ ]

   - ☐ Prefer not to say.

7. Do you have any visual or hearing impairments ?
   - ☐ No
   - ☐ Yes (visual)
   - ☐ Yes (hearing)
   - ☐ Yes (both)

     If you feel comfortable providing further details e.g. the name of your condition, please specify.

     [ ]

   - ☐ Prefer not to say.

8. What is your postcode? We will only use this information to gather publicly available consensus data on your local area.

   [ ]

   - ☐ Prefer not to say.

9. Do you identify as having a low income? (The government classifies a low income as an annual income below £20,000).
   - ☐ Yes
   - ☐ No
   - ☐ Prefer not to say

10. Do you own or have access to any of the following digital tools below? Please tick as many that apply to you.
   - ☐ Landline
   - ☐ Mobile phone (without internet)
   - ☐ Smartphone (a phone with internet connection)
   - ☐ Broadband
   - ☐ iPad or smart tablet
   - ☐ Laptop
   - ☐ Computer
   - ☐ Smart TV
   - ☐ Smart speaker (e.g. an Alexa)
   - ☐ Smart home technology (e.g. Hive)
   - ☐ Other
   - ☐ None of the above
   - ☐ Prefer not to say.

11. If you have selected any digital tools in question 10, how do you have access?
   - ☐ I own it and do not share with someone in my household.
   - ☐ I share it with someone in my household.
   - ☐ Via a family or friend.
   - ☐ Via public places e.g. libraries.

12. If you selected mobile phone or smartphone, what type of plan are you on?
   - ☐ Pay as you go
   - ☐ Contract
   - ☐ Prefer not to say.

13. What is your main reason for using technology? Please tick all the options that apply to you.
   - ☐ Work
   - ☐ Volunteering
   - ☐ To study
   - ☐ For entertainment
   - ☐ To socialise with others
   - ☐ Gaming
   - ☐ For online shopping/banking
   - ☐ Other
   - ☐ I very rarely use technology, so this question does not apply to me.
   - ☐ Prefer not to say

14. Do you feel confident when using technology?
- ☐ Not at all
- ☐ Slightly
- ☐ Somewhat
- ☐ Only if someone is helping me.
- ☐ Only with technology I am familiar with
- ☐ Fairly
- ☐ Completely

15. Please provide your name and contact details below so I can get back in touch with you to organise a chat.

Name<br><br><br>Contact details:<br><br><br>Voucher preference:

Study ID: (filled in by research team)

Follow up questionnaire

1. Is this your first-time taking part in research?
   - ☐ Yes
   - ☐ No
   - ☐ Prefer not to say

2. Have you ever used technology for health purposes? This can include video calls with a doctor or other healthcare provider, phone apps (e.g. NHS app) or wearable technology your doctor has asked you to use.
   - ☐ Yes. Please provide further details below e.g., the technology you used, and/or the purpose of using it.
   - ☐ No
   - ☐ Prefer not to say

If you answered yes to question 2 please complete questions a,b and c. If you selected no please continue to question 3.

a) What motivated you to start using the technology?
   - ☐ Family and/or friend recommended it
   - ☐ Doctor recommended it
   - ☐ Read about it online
   - ☐ Other. Please provide details below
   - ☐ Prefer not to say

b) What did you like about using the technology?
   - ☐ Prefer not to say

c) What did you find challenging about using the technology? Please tick all that apply to you.

- ☐ Finding information about the technology (to solve and issue or to learn how to use it)
- ☐ Accessing the technology or a device to use the service (e.g. a computer with a video camera for video calls, or a phone that is suitable for an app)
- ☐ Financial reasons
- ☐ Using the technology due to the complexity of the technology
- ☐ Using the technology due to small fount size or features
- ☐ Limited language translation
- ☐ Having the time to use it
- ☐ Accessing support from a family, friend or healthcare professional
- ☐ Seeing how using the technology benefits me
- ☐ None of the above – I really enjoyed using the technology
- ☐ Other. Please provide more details
- ☐ Prefer not to say

3. Would you be interested in hearing about other opportunities to take part in research?
   - ☐ Yes
   - ☐ No

   If yes, please enter your email or phone number below

COREQ (COnsolidated criteria for REporting Qualitative research) Checklist

A checklist of items that should be included in reports of qualitative research. You must report the page number in your manuscript where you consider each of the items listed in this checklist. If you have not included this information, either revise your manuscript accordingly before submitting or note N/A.

Topic	Item No.	Guide Questions/Description	Reported on Page No.
**Domain 1: Research team and reflexivity**			
Personal characteristics			
Interviewer/facilitator	1	Which author/s conducted the interview or focus group?	5
Credentials	2	What were the researcher's credentials? E.g. PhD, MD	5
Occupation	3	What was their occupation at the time of the study?	5
Gender	4	Was the researcher male or female?	5
Experience and training	5	What experience or training did the researcher have?	5
Relationship with participants			
Relationship established	6	Was a relationship established prior to study commencement?	5
Participant knowledge of the interviewer	7	What did the participants know about the researcher? e.g. personal goals, reasons for doing the research	5
Interviewer characteristics	8	What characteristics were reported about the inter viewer/facilitator? e.g. Bias, assumptions, reasons and interests in the research topic	6
**Domain 2: Study design**			
Theoretical framework			
Methodological orientation and Theory	9	What methodological orientation was stated to underpin the study? e.g. grounded theory, discourse analysis, ethnography, phenomenology, content analysis	6
Participant selection			
Sampling	10	How were participants selected? e.g. purposive, convenience, consecutive, snowball	4
Method of approach	11	How were participants approached? e.g. face-to-face, telephone, mail, email	4
Sample size	12	How many participants were in the study?	7
Non-participation	13	How many people refused to participate or dropped out? Reasons?	7
Setting			
Setting of data collection	14	Where was the data collected? e.g. home, clinic, workplace	5
Presence of non-participants	15	Was anyone else present besides the participants and researchers?	5
Description of sample	16	What are the important characteristics of the sample? e.g. demographic data, date	5,7-11
Data collection			
Interview guide	17	Were questions, prompts, guides provided by the authors? Was it pilot tested?	5
Repeat interviews	18	Were repeat inter views carried out? If yes, how many?	n/a
Audio/visual recording	19	Did the research use audio or visual recording to collect the data?	5
Field notes	20	Were field notes made during and/or after the inter view or focus group?	5
Duration	21	What was the duration of the inter views or focus group?	7
Data saturation	22	Was data saturation discussed?	5,6
Transcripts returned	23	Were transcripts returned to participants for comment and/or	n/a

Topic	Item No.	Guide Questions/Description	Reported on Page No.
		correction?	
**Domain 3: analysis and findings**			
Data analysis			
Number of data coders	24	How many data coders coded the data?	6
Description of the coding tree	25	Did authors provide a description of the coding tree?	12,16
Derivation of themes	26	Were themes identified in advance or derived from the data?	6
Software	27	What software, if applicable, was used to manage the data?	6
Participant checking	28	Did participants provide feedback on the findings?	na
Reporting			
Quotations presented	29	Were participant quotations presented to illustrate the themes/findings? Was each quotation identified? e.g. participant number	13-16
Data and findings consistent	30	Was there consistency between the data presented and the findings?	13-16
Clarity of major themes	31	Were major themes clearly presented in the findings?	13-16
Clarity of minor themes	32	Is there a description of diverse cases or discussion of minor themes?	13-16

Developed from: Tong A, Sainsbury P, Craig J. Consolidated criteria for reporting qualitative research (COREQ): a 32-item checklist for interviews and focus groups. *International Journal for Quality in Health Care*. 2007. Volume 19, Number 6: pp. 349 – 357

Once you have completed this checklist, please save a copy and upload it as part of your submission. DO NOT include this checklist as part of the main manuscript document. It must be uploaded as a separate file.
